# Assessing and improving organizational readiness to implement substance use disorder treatment in primary care: findings from the SUMMIT study

**DOI:** 10.1186/s12875-017-0673-6

**Published:** 2017-12-21

**Authors:** Allison J. Ober, Katherine E. Watkins, Sarah B. Hunter, Brett Ewing, Karen Lamp, Mimi Lind, Kirsten Becker, Keith Heinzerling, Karen C. Osilla, Allison L. Diamant, Claude M. Setodji

**Affiliations:** 1 RAND Corporation, 1776 Main Street, Santa Monica, CA 90407 USA; 2 Venice Family Clinic, 2509 Pico Boulevard, Santa Monica, CA 90405 USA; 3 UCLA Department of Family Medicine, UCLA Family Health Center, 1920 Colorado Avenue, Santa Monica, CA 90404 USA; 4 UCLA Department of Internal Medicine, Division of General Internal Medicine, 911 Broxton Avenue, Los Angeles, CA 90024 USA

**Keywords:** Implementation intervention, Organizational readiness, Evidence-based substance use disorder treatment, Primary care, Collaborative care, Care coordination, Medication-assisted treatment, Extended-release injectable naltrexone, Vivitrol®, Buprenorphine/naloxone, Suboxone®, Motivational interviewing

## Abstract

**Background:**

Millions of people with substance use disorders (SUDs) need, but do not receive, treatment. Delivering SUD treatment in primary care settings could increase access to treatment because most people visit their primary care doctors at least once a year, but evidence-based SUD treatments are underutilized in primary care settings. We used an organizational readiness intervention comprised of a cluster of implementation strategies to prepare a federally qualified health center to deliver SUD screening and evidence-based treatments (extended-release injectable naltrexone (XR-NTX) for alcohol use disorders, buprenorphine/naloxone (BUP/NX) for opioid use disorders and a brief motivational interviewing/cognitive behavioral –based psychotherapy for both disorders). This article reports the effects of the intervention on key implementation outcomes.

**Methods:**

To assess changes in organizational readiness we conducted pre- and post-intervention surveys with prescribing medical providers, behavioral health providers and general clinic staff (*N* = 69). We report on changes in implementation outcomes: acceptability, perceptions of appropriateness and feasibility, and intention to adopt the evidence-based treatments. We used Wilcoxon signed rank tests to analyze pre- to post-intervention changes.

**Results:**

After 18 months, prescribing medical providers agreed more that XR-NTX was easier to use for patients with alcohol use disorders than before the intervention, but their opinions about the effectiveness and ease of use of BUP/NX for patients with opioid use disorders did not improve. Prescribing medical providers also felt more strongly after the intervention that XR-NTX for alcohol use disorders was compatible with current practices. Opinions of general clinic staff about the appropriateness of SUD treatment in primary care improved significantly.

**Conclusions:**

Consistent with implementation theory, we found that an organizational readiness implementation intervention enhanced perceptions in some domains of practice acceptability and appropriateness. Further research will assess whether these factors, which focus on individual staff readiness, change over time and ultimately predict adoption of SUD treatments in primary care.

## Background

Several evidence-based treatments for substance use disorders (SUDs)—medications in particular, but also brief therapy—are available and appropriate for delivery in primary care settings [1–7], but are underutilized [8–11]. Primary care is thought to be well-suited to provide SUD treatment because the prevalence of alcohol use disorders and use of illicit drugs is higher among primary care patients than in the general population due to comorbidity of SUDs with other illnesses that bring people into medical care [12, 13], and most individuals visit a primary care provider at least once a year [14]. Further, expanded coverage under the Patient Protection and Affordable Care Act (PPACA) has increased the number of individuals seeking medical care in primary care clinics in United States [15, 16]. Increasing identification of SUDs and availability of SUD treatment in primary care could increase access to treatment for millions of individuals who need but never receive treatment for a number of reasons, including low perceived need for treatment, lack of readiness, SUD-related stigma, or because speciality treatment centers lack the capacity to provide timely treatment [17–20]. Despite the potential benefits, few primary care providers have integrated treatment for SUDs into their practice.

Although there is little available data on barriers to the uptake of SUD treatment by primary care providers, some of the known barriers include lack of leadership buy-in for integrating SUD care into medical practices; lack of confidence among physicians in their own or their clinic’s ability to treat SUDs; lack of adequate physician role models and access to decision support consultants; deficiencies in training and expertise in addiction treatment (i.e., workforce issues), Medicaid regulations that impede payment for the use of certain medications and same-day medical and mental health visits, and negative attitudes towards and biases against people with SUDs [21–26]. Other barriers common to the adoption of any new practice include perceived complexity and effectiveness of the new practice, perceived fit with and relevance to existing practices, and the feasibility of implementing the new practice [27], including inability to visualize how the new practice will fit into the existing workflow (i.e., lack of written protocols) [28–30]. Further, providers in primary care clinics face large workloads, imbalance between skills and increasing job demands, and lack of team support, all of which can lead to burnout and could impede organizational readiness for and implementation of new practices [31–33].

Organizational and behavioral change theories, such as Rogers’ (1995) diffusion of innovation theory [27] and Bandura’s (1977) theory of behavior change [34], posit that individuals’ perceptions of the characteristics of new practices, their self-efficacy to implement the practices, and their perceptions of the capacity of their organization to implement the practices, are critical precursors of behavior change that support implementation. These precursors are often referred to in the organizational change literature as elements of “organizational readiness” [28, 29, 35–41]. Although definitions of organizational readiness vary widely, they have in common several key constructs, such as whether an organization’s culture and climate are ready to make general changes (for example, organizations with stronger staff morale, less staff turnover, and openness to new practices in general typically are more likely to support implementation of new practices) [42–44], whether individual members view their organization as capable of change, or whether individual members are themselves prepared and willing to make a specific change or adopt a specific new practice [28, 43, 45]. Weiner et al. (2008) suggest that conceptualization of organizational readiness is most practical when it focuses on organization members’ preparedness to implement intentional change, that is, whether individuals within an organization are psychologically and behaviorally prepared and willing to implement a specific new practice. Although some studies suggest an association between various organizational readiness constructs, including readiness of individuals as well as the culture and climate of their organizations, and adoption of new practices [46–49], no studies to date have examined the readiness of individual primary care clinic providers and other clinic staff to deliver treatment for SUDs.

To prepare a federally qualified health center (FQHC) in Los Angeles, California to deliver SUD screening and treatment to patients with opioid and alcohol use disorders (OAUDs), we employed a multi-faceted implementation intervention using a cluster of implementation strategies. FQHCs are community health clinics that receive support from the U.S. government to provide primary care and other services to medically underserved populations. We tested the intervention in a FQHC because FQHCs are responsible for delivering a large proportion of publicly funded primary care treatment in the U.S. We operationalized ‘organizational readiness’ as perceptions of the acceptability, appropriateness and feasibility of practices, and intentions to adopt the practices; these constructs are considered ‘implementation outcomes’ according to implementation researchers and theorists [27, 28, 34, 45, 50] and are based on organizational and behavioral change theory [27, 34, 50]. We focused on the treatment of OAUDs because there are FDA-approved medications to treat these disorders. Our full study design, which included an RCT phase where a collaborative care service delivery intervention was added 18 months after the start of the organizational readiness intervention, is described in the study protocol paper [51].

In this article, we describe the organizational readiness intervention and present pre- to post-intervention implementation outcomes after 18 months of the intervention and prior to the RCT. Some elements of the organizational readiness intervention, such as technical assistance and training of new staff, continued throughout the RCT. We hypothesized that the organizational readiness intervention would increase provider and staff acceptance and perceptions of appropriateness and feasibility of three evidence based SUD treatments—extended-release injectable naltrexone (XR-NTX) for alcohol use disorders, buprenorphine/naloxone (BUP/NX) for opioid use disorders, and a 6-session motivational interviewing/cognitive behavioral therapy (MI/CBT)-based psychotherapy for both disorders—and greater intention to adopt these treatments among the providers (i.e., physicians for the medications and therapists for the psychotherapy). XR-NTX is effective for people with alcohol or opioid disorders and also is feasible for delivery in primary care [52–56]; BUP/NX has been proven effective for patients with opioid use disorders and is feasible for delivery in office-based settings [1–7]; and MI/CBT-based therapies have shown efficacy in reducing substance use across settings [57–60]. For this study, prescribing medical providers were trained to deliver XR-NTX for alcohol use disorders only.

## Methods

### Study setting and participants

We conducted the study at a multi-site FQHC in Los Angeles, California. The FQHC’s two largest adult-care sites, which serve approximately 20,000 low-income patients annually, were included in the study, and provide more than 106,000 primary care, specialty care, mental health, dental, and health education visits annually. Services include diagnosis, treatment, medications, follow-up care, and laboratory tests; prior to the study, the clinic did not provide screening or treatment for any SUDs. The sites were treated as a single site due to providers working across locations. Full-time staff working at the two clinics asked to participate in the survey consisted of 18 prescribing medical providers, 9 behavioral health providers, 42 medical assistants and 24 discharge coordinators (referred to as “general staff”) in Year 1, and 18 prescribing medical providers, 9 behavioral providers, 43 medical assistants and 23 discharge coordinators in Year 2.

### Organizational readiness implementation intervention

To enhance organizational readiness to provide evidence-based treatment for OAUDs, we employed multiple theory-based strategies hypothesized to increase adoption of evidence-based practices (EBP) in community organizations [28, 29, 41, 61–70]. The organizational readiness implementation intervention aimed to increase “behavioral and psychological willingness” to implement OAUD treatment in primary care among providers and staff [71].

**Fig. 1:**
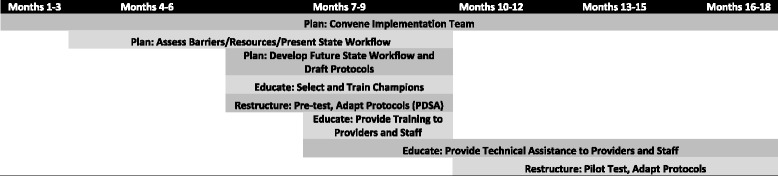
Organizational readiness implementation intervention timeline

The organizational readiness intervention consisted of six implementation strategies delivered over 18 months (see Fig. 1). The strategies fall within three key implementation process categories outlined by Powell et al. (2011): plan for change; educate at all levels; and restructure delivery systems [61].

### Process category 1: Plan for change

#### Strategy 1: Convene a collaborative researcher-clinic implementation team [28, 64]

We convened an implementation team to engage and increase buy-in [64] from clinic leadership and staff. The core team consisted of key members of the research team (the Principal Investigator and co-Investigator, with other relevant researchers joining for some meetings to provide input on special topics) and the clinic medical director and behavioral health director. The full implementation team met bi-weekly and in-person during the first 18 months of the study and once a month during the RCT. A variety of key clinic leaders were periodically invited to attend the meeting, including the clinic Chief Operating Officer, the head of the clinic’s call center (where all patient calls are received), and the nursing/medical assistant director. Core team members also conducted two briefings for the Chief Executive Officer to share updates on our implementation progress and our implementation intervention protocols—one at the organizational readiness intervention and another prior to the start of the pilot period.

#### Strategy 2: Assess barriers and resources and “present state” workflow [65, 66]

To assess barriers to and resources to deliver SUD treatment, we conducted focus groups with prescribing medical providers (*N* = 9) and behavioral health providers (*N* = 8), and semi-structured interviews with administrators (*N* = 8). Administrators included the Executive Director, Medical Director, Associate Medical Director, Behavioral Health Services Director, Nursing Supervisor, Case Manager Supervisor, Front Desk Supervisor, and Security Supervisor. To assess the “present state” workflow, we observed clinic procedures and interviewed multiple clinic leaders. We created detailed “present state” workflow diagrams to assess how SUD screening, assessment and treatment delivery would be incorporated (i.e., who would do what to whom, when, and where). In the interest of providing rapid feedback to the implementation team, we summarized key barriers identified in the focus groups and interviews and provided the summary to the team.

#### Strategy 3: Develop “future state” workflow and clinic-specific implementation protocols [28, 64]

Based on barriers identified during focus groups and interviews and on present state workflow diagrams, the research team drafted “future state” workflow and clinic-specific implementation protocols for each treatment. The implementation team then iterated until the workflow and protocols aligned with the clinic’s practices and culture. The final “future state” workflow diagram and clinic-specific implementation protocols allowed staff to visualize how (when, where and by whom) the SUD treatments would be implemented [72–74].

#### Strategy 4: Select and train “champions” [64]

We selected and trained prescribing medical provider- and behavioral health- champions. A champion is a well-respected individual within an organization who is enthusiastic about a new practice and who can serve as a role model for adopting new practices [64]. The medical director (a primary care provider) and behavioral health director (a licensed clinical social worker), along with one additional primary care provider, became champions of the SUD treatments. An addiction medicine physician led the trainings for prescribing medical providers for both of the medications. Only physicians participated in the BUP/NX training, as nurse practitioners and physician assistants were not yet authorized to prescribe this medication. An MI/CBT-expert led the psychotherapy training. After receiving training, the two primary care providers began offering the two medications to eligible patients to identify barriers and to inform development of the clinic-specific protocols [72–74]. The behavioral health director did the same with the MI/CBT-based psychotherapy.

### Process category 2: Educate at all levels

#### Strategy 5: Training and technical assistance [75–77]

We provided multiple trainings for prescribing medical providers, behavioral health providers, and general clinic staff on the SUD treatments. Trainings were tailored to specific staff roles within the organization and were as follows:

- All clinic providers, administrators, staff at all levels of the organization, and volunteers (approximately 100 staff members and clinic volunteers): A 30-min “all-staff” kick-off meeting that included an overview provided by the researchers of the significance of incorporating SUD treatment into primary care, a review of how clinic workflow would accommodate SUD treatment (i.e., the “future state” workflow), and a brief informative talk by a local, well-respected local psychologist who specializes in integration of SUD treatment in primary care;
- Prescribing medical providers: XR-NTX training consisting of 2.5 h of training for all prescribing medical providers (physicians, physician assistants, nurse practitioners) provided by an addiction medicine physician (7 participated in training during the first 18-months); “Provider’s Clinical Support System for Medication-Assisted Treatment (PCSS-MAT)” training on BUP/NX, which required completion of a 3.75-h on-line module followed by 4.25 h of in-person training by an addiction medicine physician and receipt of the X-waiver^1^ for BUP/NX prescribing (7 participated in training during the first 18-months);
- Behavioral health providers: A 1-h overview (8 participated in the first 18 months), plus 16 h of training on the MI/CBT-based brief therapy for licensed clinical social workers (LCSWs), conducted by a psychologist with expertise in MI and CBT (5 participated in the first 18 months).
- SUD care coordinators (2 care coordinators participated): 8 h of training on care coordination and motivational interviewing techniques provided by the researchers and the MI/CBT expert. The two care coordinators in this study were paraprofessionals who also had other responsibilities at the clinic;
- Nurse practitioners and physician assistants (3 and 2, respectively, participated in training during the first 18 months): 1 h of training on administering XR-NTX conducted by an addiction medicine physician;
- Clinic pharmacists and pharmacy technicians (4 and 3, respectively, participated): 1-h of training on medications to be provided to patients and pharmacy procedures for administering XR-NTX (BUP/NX was not provided by the clinic pharmacy), conducted by an addiction medicine physician.
- Medical assistants, call center staff, discharge coordinators: 1 h of training on SUD screening and referral procedures with two booster trainings and individual trainings for volunteers, who also conducted screenings, conducted by research staff.

We also provided ongoing technical assistance consisting of access to the addiction medicine physician and MI/CBT expert and ongoing support and monitoring of screening, referral and care coordination by research staff.

### Process category 3: Restructure delivery systems

#### Strategy 6: Pre-test protocols using plan-do-study-act (PDSA) cycles, pilot test protocols, complete protocol adaptation

Soon after the treatment protocols were drafted, the champions began implementing the three treatments. That is, the two medical provider champions began to deliver XR-NTX and BUP/NX to eligible, interested patients and the behavioral health champion began providing the MI-based psychotherapy to eligible, interested patients. Although the designated champions initiated delivery, medication administration also involved nurse practitioners (to deliver XR-NTX injections) and pharmacy staff (to dispense and track XR-NTX). Throughout this process, the implementation team made iterative adaptations to the protocols using plan-do-study-act (PDSA) cycles [67–70], revising the protocols as feedback on implementation was received, and then repeating the cycle. PDSA cycles offer a structured approach to engaging staff in making iterative, feedback-based changes in service delivery [67, 68]. Adaptation of protocols helps ensure the fit of protocols within the organization and can lower resistance from individuals who will be affected by the change in delivery [29]. Of note, prior to the study, the clinic did not conduct screening for SUD, a sizable barrier to implementing treatment. Because the clinic needed to implement screening in order to identify patients with SUD, we included SUD screening in the future state clinic workflow and assisted in its adoption in a similar fashion as the other practices—we began with a screening protocol and tested and revised it using PDSA cycles. After all staff members were trained and protocols were developed (after 10 months of the organizational readiness intervention), we conducted an 8-month pilot study that involved screening and treating patients using draft protocols. Final protocol adaptations were made following the pilot.

### Data collection procedures and measures

#### Procedures

For the present analysis, we used data from staff surveys conducted before the organizational readiness intervention was executed and 18-months later (and prior to the RCT) to assess changes in acceptability, perceptions of appropriateness and feasibility of, and intention to adopt SUD treatment during this period. The survey included previously developed measures, modifications of previously developed measures, as well as locally developed items. Surveys were web-based; for providers without access to clinic e-mail, we distributed paper and pencil surveys.

#### Measures

**(1)** ***Acceptability***. Acceptability refers to satisfaction with the *complexity or ease of use* of a new practice and its effectiveness, or credibility [50]. To measure acceptability we adapted items from Moore and Benbasat’s validated instrument [78] which measures items that parallel elements of successful diffusion of new practices [27]. Within the *acceptability* domain, we measured *ease of use* perceptions among prescribing medical providers (a two-item scale for each of the two medications) and behavioral health providers (the same two-item scale but pertaining to the MI/CBT-based psychotherapy). The two scale items were “*overall, I believe that extended-release injectable naltrexone [or other EBP] is easy to use,*” *and “extended-release injectable naltrexone [or other EBP] is clear and understandable.”* Response options ranged from 1, Extremely Disagree, to 7, Extremely Agree (α = 0.96). We also included items from the National Center for Addiction and Substance Abuse’s (CASA) National Survey of Primary Care Physicians and Patients on Substance Abuse [79]) that capture providers’ opinions about the *effectiveness* of SUD EBP (one item per EBP) (response options ranged from 1, Not Effective, to 4, Very Effective), as well whether providers find it difficult to discuss SUDs with their patients (two items per EBP) (response options ranged from 1, Very Difficult, to 4, Not at all Difficult). **(2)** ***Appropriateness***. Appropriateness refers to the compatibility of each EBP with current practices [50]. To measure compatibility of the EBPs with current practices we adapted an item from Moore and Benbasat for each EBP [78]. This item was “*I think buprenorphine/naloxone [or other EBP] will fit with the way I like to work.”* Response options ranged from 1, Extremely Disagree, to 7, Extremely Agree. We also used locally-developed items to ask about whether providers and general staff believed SUDs could be effectively treated in primary care and the clinic itself, and also asked about fit with the clinic’s mission and values (three items). Response options ranged from 1, Strongly Disagree, to 5, Strongly Agree. **(3)** ***Feasibility***. Feasibility is the actual fit of an EBP within an organization [50]. We asked about feasibility using items from CASA’s National Survey of Primary Care Physicians and Patients on Substance Abuse [79] that capture how prepared providers feel about identifying patients with alcohol use disorders (AUD) and opioid use disorders (OUD) (one item for AUD, one for prescription OUD, and one for street OUD/heroin) (response options ranged from 1, Not at all Prepared, to 5, Very Prepared). **(4)** ***Intent and/or willingness to adopt the EBP***. To measure intention or willingness to adopt, we used the three-item “demonstrability” scale from Moore and Bensabet [78], which present statements such as “*I believe I can communicate to others the consequences of using extended-release injectable naltrexone [or other EBP].*” We also asked providers questions about whether they were willing to consider using each of the EBPs in their practice (e.g.*, I would consider using buprenorphine/naloxone in my practice*). Note that for all variables that asked prescribing medical providers about medications, our pre-intervention survey items combined the two medications (XR-NTX and BUP/NX) and referred to “medication-assisted treatment (MAT) for OAUDs,” as providers were not familiar with each medication. Our instructions for these items described the two medications and explained the indications of each; we use this measure as a proxy for the pre-intervention assessment of each medication. For our post-intervention measures, we asked questions about each medication separately. Post-intervention, all prescribing medical providers were asked about XR-NTX, and only physicians (not nurse practitioners or physician’s assistants) were asked about BUP/NX because at the time of the survey only physicians were authorized to prescribe this medication.

#### Analysis

To measure changes between the two time points we conducted Wilcoxon signed rank tests. To adjust *p*-values for multiple tests, we used Benjamini and Hochberg false discovery rate (FDR) correction [80]. We examined responses by type of clinic staff. Due to staff turnover and post-intervention non-response, the sample size varied across variables and percentages are based on the total number of participants who responded both pre- and post-intervention. Due to the very small number of behavioral health providers who completed surveys at both time periods, we do not report on changes in behavioral health providers’ perceptions.

## Results

### Participant characteristics

Fifteen of 18 (83%) prescribing medical providers, 8 of 9 (89%) behavioral health providers and 46 of 66 (70%) general clinic staff (medical assistants and clinic coordinators) completed the year 1 survey and 16 of 20 (80%) prescribing medical providers, 8 of 9 (89%) behavioral health providers and 46 of 66 (70%) general clinic staff completed the year 2 survey. Participating staff were mostly female (84%) and Hispanic (70%). More than half of the staff who responded (52%) had been in their current position at the clinic for more than 10 years (see Table 1).

**Table 1:** Staff characteristics

		All staff		Medical providers		Behavioral health providers		Non-provider staff	
	N	Missing	Mean/%	N	Mean/%	N	Mean/%	N	Mean/%
Age	66	3	44.41	15	45.13	8	44.50	43	44.14
Female	56	13	83.58	13	86.67	5	62.50	38	86.36
Highest Education		2							
< High School/High School	9		13.43	0	0.00	1	12.50	8	18.18
Associates/Bachelor Degree	13		19.40	0	0.00	1	12.50	12	27.27
Doctoral	13		19.40	12	80.00	1	12.50	0	0.00
Masters	9		13.43	3	20.00	5	62.50	1	2.27
Other	9		13.43	0	0.00	0	0.00	9	20.45
Some College	14		20.90	0	0.00	0	0.00	14	31.82
Time at Current Position		2							
3–10 Years	19		28.36	4	26.67	3	37.50	12	27.27
< 3 Years	13		19.40	4	26.67	1	12.50	8	18.18
> 10 Years	35		52.24	7	46.67	4	50.00	24	54.55
Race/ethnicity		16							
White	12		22.64	9	75.00	1	16.67	2	5.71
Black	2		3.77	1	8.33	0	0	1	2.86
Asian	1		1.89	1	8.33	0	0	0	0
Hispanic/Latino	37		69.81	1	8.33	5	83.33	31	88.57
Other	1		1.89	0	0	0	0	1	2.86

### Pre-post organizational readiness intervention results

#### Acceptability

As shown in Table 2, prescribing medical providers’ perceptions of the acceptability of medication-assisted treatment were fairly low across most domains prior to the intervention execution, with mean scores for ease of use at 3.05 (all prescribing providers) and 2.94 (physicians only), on a 7-point scale (7 = strongly agree with statements about ease of use); mean effectiveness scores ranging from 2.33 to 2.9 on a 4-point scale (4 = very effective); and difficulty of discussing alcohol use and opioid use with patients at 3.25 and 2.67, respectively, on a 4-point scale (4 = not at all difficult).

**Table 2:** Changes in organizational readiness to deliver SUD treatment in primary care

	Pre-Intervention			Post-Intervention			Difference		
	Mean	SD	Range	Mean	SD	Range	Mean	SD	*P*-value^^^
Acceptability									
*Prescribing Medical Providers*									
Ease of Use (Extremely Disagree = 1; Extremely Agree = 7)									
Ease of Use of XR-NTX (*N* = 11)^†^	3.05	1.29	1–4.5	4.77	1.23	3–7	1.73	1.69	0.012*
Ease of Use of BUP/NX (*N* = 9)^†^	2.94	1.40	1–4.5	2.50	1.09	1–4	−0.44	1.42	0.500
Effectiveness (Not Effective = 1; Very Effective = 4)									
… medical treatments for alcohol use disorders (*N* = 9)	2.33	0.71	1–3	3.22	0.44	3–4	0.89	0.78	0.031
… medical treatments for opioid use disorders (*N* = 9)	2.44	0.73	1–3	3.00	0.5	2–4	0.56	0.73	0.125
… mental health treatments for alcohol use disorders (*N* = 10)	2.90	0.74	2–4	3.20	0.42	3–4	0.30	0.67	0.193
… mental health treatments for opioid use disorders (*N* = 10)	2.90	0.74	2–4	3.10	0.57	2–4	0.20	0.63	0.343
Difficulty discussing … (Very Difficult = 1; Not at all Difficult = 4)									
… alcohol abuse with your patients (*N* = 12)	3.25	0.45	3–4	3.25	0.75	2–4	0.00	0.60	1.000
… opioid abuse with your patients (*N* = 12)	2.67	0.65	2–4	3.08	0.79	2–4	0.42	1.00	0.175
Appropriateness									
*Prescribing Medical Providers*									
Compatibility of SUD Treatment with Primary Care (Strongly Disagree = 1; Strongly Agree = 5)									
Substance use disorders can be effectively treated in a primary care setting (*N* = 12)	3.00	0.60	2–4	4.25	0.87	2–5	1.25	0.97	0.006*
Substance use disorders can be effectively treated at [THIS CLINIC] (*N* = 12)	2.83	0.83	1–4	3.17	0.83	2–4	0.33	0.98	0.398
Providing medications to patients with alcohol or opioid use disorders fits with [THIS CLINIC’S] mission and goals (*N* = 12)	3.17	1.19	1–5	3.17	0.83	2–4	0.00	1.21	1.000
Providing counseling to patients with alcohol or opioid use disorders fits with [THIS CLINIC’S] mission and goals (*N* = 12)	4.42	0.67	3–5	3.67	0.98	2–5	−0.75	0.62	0.002*
Compatibility with Current Practice (Extremely Disagree = 1; Extremely Agree = 7)									
Perceived Compatibility of XR-NTX with current practices (*N* = 11)^†^	3.36	1.79	1–7	4.77	1.33	2–7	1.41	1.14	0.004*
Perceived Compatibility of BUP/NX with current practices (*N* = 8)^†^	3.13	2.03	1–7	2.63	1.38	1–5	−0.50	1.34	0.375
*General Clinic Staff*									
Compatibility of SUD Treatment in Primary Care (Strongly Disagree = 1; Strongly Agree = 5)									
Substance use disorders can be effectively treated in a primary care setting (*N* = 35)	3.17	1.01	1–5	4.06	0.76	1–5	0.89	1.08	<.0001**
Substance use disorders can be effectively treated at [THIS CLINIC] (*N* = 35)	3.17	0.98	1–5	3.86	0.77	2–5	0.69	1.08	<.0001**
Providing medications to patients with alcohol or opioid use disorders fits with [THIS CLINIC’S] mission and goals (*N* = 35)	2.94	0.97	1–5	3.89	0.76	2–5	0.94	1.24	<.0001**
Providing counseling to patients with alcohol or opiate use disorders fits with [THIS CLINIC’S] mission and goals. (*N* = 36)	3.72	0.94	1–5	3.67	1.07	2–5	−0.06	1.43	0.817
Feasibility									
*Prescribing Medical Providers*									
Feel prepared to … (Not at all Prepared = 1; Very prepared = 4)									
… identify patients with alcohol use disorders (*N* = 12)	3.42	0.67	2–4	3.92	0.29	3–4	0.50	0.52	0.031
… identify patients who are using illegal opiates such as heroin (*N* = 11)	3.27	0.65	2–4	3.45	0.52	3–4	0.18	0.60	0.625
… identify patients who are misusing (*N* = 12)or abusing prescription opioids (*N* = 12)	3.00	0.60	2–4	3.33	0.65	2–4	0.33	0.49	0.125
Intent/willingness to adopt ebp									
*Prescribing Medical Providers*									
Perceived demonstrability (Extremely Disagree = 1; Extremely Agree = 7)									
… of XR-NTX (*N* = 11)^†^	4.21	1.20	1.67–6.33	4.85	0.91	4–7	0.64	1.11	0.086
… of BUP/NX (*N* = 9)^†^	4.11	1.28	1.67–6.33	3.30	1.31	1–5.33	−0.81	1.11	0.058
Would consider using in current practice (Extremely Disagree = 1; Extremely Agree = 7)									
I would consider using XR-NTX in my practice (*N* = 11)	4.09	1.87	2–7	5.55	1.13	4–7	1.45	1.81	0.037
I would consider using BUP/NX in my practice (*N* = 10)	3.80	1.69	2–7	4.30	2.16	1–7	0.50	1.72	0.469

^†^Pre-intervention measures combined the two medications into one question asking about MAT for OAUD; all prescribing providers were asked about XR-NTX and only physicians were asked about BUP/NX

^^^The Benjamini and Hochberg false discovery rate (FDR) correction was used to assess significance given the multiple testing. *P*-values reported in this column are those prior to FDR correction

*Significance at *p* < 0.05 after FDR correction

**Significance at *p* < 0.01 after FDR correction

Among all prescribing providers, ease of use of XR-NTX for alcohol use disorders changed significantly (*p* < .05) from pre- to post- intervention, with mean scores changing from 3.05 pre- to 4.77 post- intervention, with standard deviations of 1.29 and 1.23, respectively. Changes in perceptions of ease of use of BUP/NX did not change significantly, and mean scores were lower in after the intervention than XR-NTX ease of use scores. Perceptions of the effectiveness of medication for the treatment of alcohol use disorders improved, but changes were not significant after the FDR adjustment for multiple statistical tests. Perceptions of ease of use of BUP/NX and effectiveness of medication for opioid use disorders did not change significantly, but were similar to post-intervention mean scores for XR-NTX effectiveness. Prescribing medical providers’ perceptions of the effectiveness of mental health counseling for either disorder was over the mid-point at both time points and did not change significantly.

### Appropriateness

In the appropriateness domains, prescribing medical providers’ perceptions of the compatibility of aspects of SUD treatment with primary care varied pre-intervention by the type of treatment, with the mean score for the fit of counseling for patients with alcohol and opioid use disorders at the clinic at 4.42 (the highest), the mean score for whether SUDs could be treated at this clinic at 2.83 (the lowest), on a scale from 1 to 5 (5 = strongly agree with statements about appropriateness in primary care). Among general clinic staff, pre-intervention scores were similar, with scores ranging from 2.94 (fit of providing medications to patients with OAUDs at this clinic) to 3.72 (fit of providing counseling to patients with OAUDs with clinic mission and goals), also on a 5-point scale. With regard to prescribing medical providers’ perceptions of compatibility of MAT with current practices, mean scores were fairly low—3.36 among all prescribing providers and 3.13 among physicians only, on a scale from 1 to 7 (7 = strongly agree with statements about compatibility).

Prescribing medical providers agreed more post-intervention that SUDs could be treated in primary care settings (in general), with mean scores changing significantly (*p* < .01) on a scale from 1 to 5, with a 5 indicating strong agreement (M = 3.00, SD = .60 pre-intervention; M = 4.26, SD = .87 post-intervention). However, views about whether SUDs could be treated at this clinic did not change. Post-intervention, prescribing providers also thought XR-NTX was more compatible with their current practices than they did pre-intervention, with statistically significant (*p* < .01) changes in mean scores on a scale from 1 to 5, with 5 indicating a high degree of compatibility (M = 3.36, SD = 1.79 pre-intervention; M = 4.77, SD = 1.22, post-intervention); opinions of compatibility of BUP/NX with current practices, did not change significantly pre- and post-intervention. Prescribing medical providers’ ratings of the fit of counseling for SUDs in primary care with the clinic’s mission and goals were significantly (*p* < .01) lower post- intervention on a scale from 1 to 5, with a 5 indicating strong agreement with a statement about fit (M = 4.42, SD = .67 pre-intervention; M = 3.67, SD = .98 post-intervention).

Among general clinic staff, views about the appropriateness of SUD treatment changed significantly pre- to post-intervention (*p* < .01). Post-intervention, staff agreed more with statements suggesting that SUDs could be treated in primary care settings on a scale from 1 to 5, with 5 indicating strong agreement with the statement (M = 3.17, SD = 1.01 pre-intervention; M = 4.06, SD = .76 post-intervention), that SUDs could be effectively treated at this clinic (M = 3.17, SD.98 pre-intervention; M = 3.86, SD = .77 post-intervention), and that providing medications to patients with alcohol or opioid use disorders fit the mission and goals of the clinic (M = 2.94, SD = .97 pre-intervention; M = 3.89, SD = .76 post-intervention). General clinic staff perceptions that providing counseling to patients with alcohol and opioid use disorders fit the clinic’s mission and goals was initially positive and did not change pre- to post-intervention.

### Feasibility

Before the organizational readiness intervention, prescribing medical providers’ mean scores on feeling prepared to identify patients with alcohol, heroin and prescription opioid use disorders were 3.0, 3.27 and 3.42, respectively, on a scale from 1 to 4 (4 = very prepared). Post-intervention, prescribing medical providers felt more prepared than at baseline to identify patients with alcohol disorders, but these changes were not statistically significant after the FDR correction.

### Intention to adopt

Pre-intervention, prescribing medical providers’ scores were similar for perceived demonstrability, with mean scores of 4.21 (all prescribing providers, XR-NTX) and 4.11 (physicians, BUP/NX), on a scale from 1 to 7 (7 = strongly agree with statements of demonstrability). With respect to whether providers would consider using each medication in their practice, mean scores were 4.09 for XR-NTX and 3.80 for BUP/NX. Post-intervention, prescribing medical providers were more willing to use XR-NTX in their practice but these changes were not statistically significant after the FDR correction. Willingness to use BUP/NX did not change significantly.

## Discussion

We hypothesized that a multi-component implementation intervention aimed at planning, educating providers and staff, and restructuring the care delivery system would lead to greater organizational readiness, measured by four implementation outcome domains: improved perceptions of acceptability, appropriateness, feasibility and intention or willingness to adopt evidence based treatments for OAUDs; these hypotheses were partially supported. Specifically, convening a researcher-clinic implementation team, assessing barriers, resources and workflows, creating future state workflows and protocols tested by champions and in a clinic-wide pilot, providing training and technical assistance to all providers and general clinic staff, adapting workflows and protocols to meet clinic needs, improved perceptions of the appropriateness of treating SUD in primary care among both prescribing medical providers and the general clinic staff and perceptions of the acceptability of XR-NTX among prescribing medical providers. Immediately post-intervention, prescribing medical providers showed greater changes in their perceptions of ease of use and compatibility of XR-NTX with current practice, but no changes with respect to perceptions of BUP/NX ease or compatibility. Prescribing medical providers’ intention to adopt XR-NTX also increased after the organizational readiness intervention, but changes were not significant after the FDR correction. General clinic staff had significant improvements in their opinions about the appropriateness of SUD treatment in primary care on three of three measures – that SUDs can be effectively treated in primary care, that SUDs can be effectively treated at this clinic, and that providing medications for alcohol or opiate use disorders fits the clinic’s mission and goals. These results suggest that the organizational readiness intervention was successful at changing at least some important implementation outcomes, which are theoretically associated with the adoption of new practices [50].

Prescribing medical providers’ opinions changed significantly in a negative direction with respect to their belief that providing counseling to patients with alcohol and opioid use disorders fit with the clinic’s mission and goals. This may have been because during the organizational readiness intervention period, prescribing medical providers incorrectly perceived that the question about counseling pertained to prescribing medical providers providing counseling, instead of behavioral health providers. Interestingly, opinions of general clinic staff about the fit of providing counseling for opioid and alcohol use disorders did not change significantly, but the mean score was already higher (i.e., supporting the idea that counseling was a good fit) than the mean scores of the variables that did change. Although general clinic staff will not directly “adopt” the SUD treatments, they do play an important role in screening patients and making appointments for patients seeking SUD treatment; implementation theory suggests that ultimately opinions and buy-in of staff at all levels of the organization could affect overall adoption of SUD treatment at the clinic [29].

Although our hypotheses generally were supported, the findings were somewhat nuanced. First, although prescribing medical providers and general staff opinions improved with regard to the appropriateness of providing SUD treatment in primary care in general, prescribing medical providers’ opinions about the appropriateness of providing SUD treatment at their particular clinic did not change significantly. Further, and perhaps related to this, while the findings suggest that the intervention we employed may have improved prescribing medical providers' readiness in the “acceptability” and “appropriateness” domains to deliver XR-NTX to patients with alcohol use disorders, the intervention did not improve readiness in those domains for the use of BUP/NX to treat opioid disorders, despite providers having treatment protocols that were adapted for their setting and extensive training. It is possible that views about treating patients with SUDs in general and about using BUP/NX to treat patients with opioid use disorders could improve with hands-on experience treating patients. Further, although the change was not statistically significant, prescribing medical providers’ opinions about the demonstrability of BUP/NX actually changed in a negative direction after the 18 months of the intervention. While we do not know the reasons for the perceived differences between XR-NTX and BUP/NX, it may be that the need to get a DEA waiver to prescribe BUP/NX, the DEA requirement that providers maintain a list of all patients being prescribed BUP/NX and participate in DEA audits, and that the clinic pharmacy does not include narcotics on the clinic formulary, influenced perceptions of BUP/NX’s acceptability and appropriateness for the clinic. Further, it is also possible that opinions will change after implementation of the service delivery intervention, with champions continuing to prescribe BUP/NX and more patients with OAUDs being referred to prescribing medical providers for treatment.

Importantly, prescribing medical providers’ views of the feasibility of and their intention to adopt either XR-NTX or BUP/NX did not change after we adjusted for multiple statistical tests, although there was a trend towards providers perceiving XR-NTX as more feasible and being willing to adopt this practice. The lack of statistical significance may be because of the small sample size and need to correct for multiple comparisons, or because additional interventions are needed to change these readiness outcomes.

Of note, although this study was designed to increase the clinic’s capacity (i.e., infrastructure) to deliver SUD treatment, which can affect adoption of new practices [45, 81, 82], we did not objectively measure organizational capacity. However, providers’ perceptions of whether the treatments are acceptable and appropriate may reflect perceptions of capacity. As noted by Weiner et al. (2008), to be motivated and willing to implement a new practice, individuals must believe that they and their organizations are capable of delivering an intervention [28]. As health care organizations attempt to integrate SUD and other EBPs, it may be important for implementation interventions to explicitly address capacity as part of improving organizational readiness. Health care organizations must also consider the feasibility of implementing the organizational readiness intervention itself, and some adaptation (for example, in meeting frequency, number of providers invited to attend training, length of training) may be needed to fit individual settings.

This study has some limitations. First, we used a pre-post design, at a single FQHC, with no control group. Therefore, our findings cannot be solely attributed to the organizational readiness intervention, as there may have been other changes in or outside of the clinic or external environment that influenced findings. Generalizability to other FQHCs and other primary care settings is limited, as organizational culture and patient populations may differ across different types of primary care practices. Future study of the effects of the intervention on organizational readiness, including measures of organizational functioning (e.g., turnover, morale), in a larger, randomized sample of primary care clinics is needed. We describe our findings using central tendency statistics (means and standard deviations) for ease of interpretation, but caution should be used in interpreting the results because of the small sample sizes*.* Because there are few validated measures of implementation outcomes, we included some locally developed items and items from scales that have not been validated. In addition, in our pre-intervention survey, the two medications were combined and compared with follow-up measures that asked about each medication separately. We tested a bundled intervention that comprised of several different implementation strategies designed to enhance organizational readiness, and cannot separate the effects of each strategy. Finally, because we believe that perceptions of acceptability, appropriateness and feasibility, as well as intent to adopt continue to improve as staff and providers see patients benefitting from receiving treatment for their OAUD, we do not view these outcomes as final measures of the effectiveness of the organizational readiness intervention or as final indicators of the readiness of prescribing medical providers to adopt either medication.

## Conclusions

We found that an organizational readiness intervention consisting of multiple theoretically grounded implementation strategies aimed at the entire organization, improved some implementation outcomes related to integrating treatment for OAUDs in primary care, but not all. There were differences in prescribing medical providers' perceptions of XR-NTX and BUP/NX, and providers generally saw treating patients with alcohol use disorders with XR-NTX as more acceptable and appropriate than treating patients with opioid use disorders with BUP/NX immediately after 18-months of the intervention. Additional work may be needed to prepare primary care providers to treat patients with OUDs and to  adopt BUP/NX. These results demonstrate the value of incorporating several implementation strategies to build support for implementation of SUD treatment in primary care settings.

## Abbreviations

AUD: Alcohol use disorder
BUP/NX: Buprenorphine/Naloxone
CASA: Center for addiction and substance abuse
CBT: Cognitive behavioral therapy
DEA: Drug enforcement agency
EBP: Evidence-based practice
FDA: Food and drug administration
FQHC: Federally Qualified Health Center
HSPC: Human subject protection committee
MI: Motivational interviewing
NIDA: National institute on drug abuse
OAUD: Opioid and alcohol use disorders
OUD: Opioid use disorder
PCSS-MAT: Provider’s clinical support system for medication-assisted treatment
PDSA: Plan-Do-Study-Act
PPACA: Patient protection and affordable care act
RCT: Randomized controlled trial
SUD: Substance use disorder
SUMMIT: Substance use, motivation and medication integrated treatment
XR-NTX: Extended-release Naltrexone

## Footnotes

1 In the U.S., medical providers are permitted to dispense or prescribe approved Schedule III, IV, and V narcotic medications (i.e., those that have a lower risk for abuse, like buprenorphine/naloxone) in settings other than an opioid treatment program (OTP). In order to prescribe or dispense buprenorphine, medical providers must qualify for an “X-waiver” which includes completing 8 h of required training and applying for the wavier with the drug enforcement agency (DEA) [83].
